# A vertical-horizontal approach to examine social inequalities in early onset type 2 diabetes in the German workforce through occupational sector, education and income

**DOI:** 10.1038/s41598-025-95326-x

**Published:** 2025-03-26

**Authors:** Batoul Safieddine, Siegfried Geyer, Stefanie Sperlich, Julia Grasshoff, Johannes Beller

**Affiliations:** Medical Sociology Unit, Hannover Medical School, OE 5420 Carl-Neuberg-Street 1, 30625 Hannover, Germany

**Keywords:** Social inequalities, Occupational group, Early onset T2D, Intersectionality, Germany, Claims data, Diseases, Endocrinology, Health care, Risk factors

## Abstract

Early onset type 2 diabetes (T2D) is increasingly recognized as a significant public health concern, leading to more severe complications and a greater decline in quality of life compared to T2D diagnosed later in life. This can have a profound impact on the workforce. Social status—whether assessed vertically through levels of income, education or job position or horizontally through occupational groups—can play a critical role in the risk of developing early onset T2D. While research focusing on vertical socioeconomic inequalities related to T2D is abundant, there is currently no study that combines both vertical and horizontal perspectives to explore vulnerable groups. We aim to combine the vertical and horizontal approaches to examine vulnerable groups within the employed population regarding early onset T2D. Using data from the largest statutory health insurance provider in the state of lower Saxony, Germany for the year 2019 “Allgemeine Ortskrankenkasse Niedersachsen” (AOKN), we examined education and income inequalities in early onset T2D among nine occupational sectors using logistic regression analyses (*N* = 365059). Age and gender adjusted prevalence rates as illustrated by predicted probabilities were displayed to compare rates of early onset T2D among different education and income levels and occupational groups. Regression tree analysis was used to examine intersectionality between the vertical (levels of income and education) and the horizontal (occupational sector) dimensions in order to determine the most vulnerable groups. Both vertical and horizontal inequalities in early onset T2D exist within the employed population. On the one hand, disparities in education and income were present across various occupational sectors. On the other hand, significant differences in T2D prevalence could be observed within the same education and income levels across different sectors. Notably, affiliation to occupational sector was the primary factor influencing vulnerability to early onset T2D, followed by educational attainment. Individuals with low education working in the “Transport, logistics, protection and security” and “Health, social work, teaching, and education” sectors were among the most vulnerable. It is important to simultaneously examine both vertical and horizontal dimensions of inequalities to identify vulnerable groups within the workforce. Future research should adopt this approach while also exploring other populations and health outcomes.

## Introduction

Studies on associations of social inequalities and health are usually conducted using education, income, and/or occupation as indicators of social differentiation^1,2^. Some more recent studies also used wealth^3^, but compared with the large number of studies using the three first mentioned indicators, wealth, typically measured by ownership of money or financial assets, has been less commonly used to represent social inequality. Moreover, evidence suggests its effects on health are relatively limited^4^. Irrespective of the outcome chosen, each one of these indicators, either considered alone or in combination, turned out to be associated with specific diagnoses^1,2,5,6^, or general subjective measures of health^7^. Studies using the abovementioned indicators are explicitly or implicitly bound to a concept of *vertical* inequalities, i.e., there is a hierarchy where those at the top have the highest ranks for education, income and occupational position, while those at the other end are holding the lowest positions. Doing so, occupational positions with different work contexts that influence health behaviour and shape metabolic risk profiles can be subsumed under general terms, thus making heterogeneous categories conceptually comparable. One silent implication of the vertical approach is the assumption that positions subsumed under the same level of education, occupation or income are in some way homogeneous. Whether this assumption is justified is open to empirical proof. Taking the arguments together, there are reasons to assume that a vertical approach to social differentiation may be too simplistic as it ignores the between-variation of the same category-levels.

Nevertheless, while examining vertical socioeconomic inequalities is crucial to define vulnerable population subgroups, this approach needs to be complemented by a *horizontal *stratification that compares specific characteristics of occupations with respect to their associations with indicators of impaired health and/or disease. The concept of “horizontal inequality” emerged in the 1980s to address the need for social structure analysis to include dimensions beyond traditional indicators like occupational position, education, and income^8,9^. By the 1990s, this led to multidimensional models explaining health inequalities more comprehensively, especially in terms of gender differences^10–12^. However, socio-epidemiological research still often relies on class-specific views, underutilizing broader, more nuanced analyses, especially with respect to occupational differences. The number of studies comparing different occupations remains to be relatively low^13–15^. A study from Belgium compared occupational groups^16^, but also this study was rather bound to the mainstream approach described above. An Indian study focussed on employees of call centers, but did not compare them with other occupations^17^. Moreover, a study from the US compared different occupational groups with respect to cardiovascular health status, displaying occupational group disparities in physical activity, blood pressure and body mass index^18^. Furthermore, a recent German study examined determinants of nonparticipation in workplace health promotion measures among four broad categories of occupational sectors^19^. Nevertheless, performing analyses of specific occupations would make it possible to identify specific characteristics of work or strain, which in turn may facilitate introducing specific preventive or health-promoting measures. However, examining only occupations falls short of covering the complexity of occupations as it ignores variations within occupational categories.

The most comprehensive way of taking working conditions into account may be to combine the *vertical* and the *horizontal* approach. The result is a matrix that includes different positions within specific occupational groups. To our knowledge, this approach has not yet been used in studies aiming to investigate inequalities in health and define vulnerable groups. A main reason may be the large case numbers necessary to depict hierarchical positions as the “classic approach” within a broad range of occupational groups. In surveys, this may be particularly difficult to implement due to the risk of obtaining small and uninterpretable case numbers. With our study we want to pursue a ***vertical-horizontal approach ***to social inequalities in health by using data from a large German statutory health insurance provider that covers the largest part of the social structure in Germany^20^. In this study, we consider an important measure of health impairment as outcome, namely **type 2 diabetes** (T2D), over different occupational groups and social positions.

T2D is a chronic metabolic disorder characterized by high blood sugar levels resulting from the body’s inability to properly use or produce insulin. Unlike Type 1 Diabetes (T1D), which is typically diagnosed in childhood and is primarily caused by an autoimmune response that destroys insulin-producing cells in the pancreas, T2D usually develops in adulthood and is strongly associated with lifestyle factors, including poor diet, physical inactivity, and excessive body weight. While T1D requires lifelong insulin therapy, T2D is often managed with oral hypoglycemic medications, lifestyle changes, and insulin therapy only in more advanced cases^21^. Key risk factors for T2D include an unbalanced diet high in processed foods, sedentary behaviour, smoking, lack of physical activity and obesity^22,23^. The onset of T2D can be greatly influenced by living environments such as occupations that can promote risk factors like long hours of sedentary work, poor dietary habits, stress and limited access to health-promoting resources^24–29^. These environmental factors could intersect with socioeconomic conditions and determine risks for T2D, making certain subgroups of the working population particularly vulnerable.

T2D has been one of the most common chronic diseases with evidence on drastically rising trends and burden^30^. Individuals with T2D are becoming more morbid over time as depicted by a significant temporal rise in T2D comorbidities and severity^31,32^and increasing disability worldwide^30^. While this applies to all population subgroups^33^, the working population, representing about 77% of individuals at working age in Germany, is a key group for managing and preventing T2D. This is especially relevant given the rising trend of sedentary lifestyle with prolonged sitting time^34,35 ^and increasing obesity rates in middle-aged individuals^36^. In fact, changes in work environments, such as the shift to more digitalization and remote work, may contribute to higher risks for non-communicable diseases like T2D. Moreover, the work environment can greatly influence the metabolic risk profiles of individuals. Level of workplace physical activity^37^, stress^29,38^, shift work^28,39 ^and exposure to chemicals^40^ are examples of workplace exposures that shape metabolic risk profiles and act as risk factors for T2D and other chronic diseases. Therefore, it is crucial to focus on the working population and identify specific vulnerable occupational subgroups for targeted interventions.

Yet, while many studies have examined vertical socioeconomic inequalities in T2D as depicted by educational level, income and/ or occupational position^41,42^, studies examining occupational group differences in the risk of T2D are scarce, and to our knowledge, no studies exist that combine vertical and horizontal social inequalities in T2D in terms of occupational groups. Recently, we initiated investigating occupational group differences in T2D by examining the rates and trends of T2D among nine occupational sectors stratified by two age groups. The study identified different vulnerability levels among different occupational sectors. Moreover, the study showed that in younger working individuals (18–45 years), the prevalence of T2D has significantly increased between 2012 and 2019, demonstrating younger working individuals as an important vulnerable group that needs to be investigated more deeply^43^. In fact, available evidence points towards an increasing risk for T2D being diagnosed at an early age^44,45^, which is highly relevant within the background of increasing overweight and obesity rates in younger individuals^36,46^. Individuals with early onset T2D are more likely to experience severe complications earlier in life, which also accompany them for a longer duration given the rising life expectancy in T2D^47–49^. Moreover, early onset of T2D is also associated with mental health challenges like depression and anxiety which might be due to the burden of managing a chronic illness during the most active years of life^50^.

As a matter of fact, early-onset T2D is a growing public health issue associated with economic and healthcare burden^51^. Thus, especially among working individuals, early onset T2D is an important outcome to investigate not only because occupational contexts can shape the metabolic risk profiles of individuals, but also due to its psychosocial impact and potential effects on productivity, work cessation and the economic burden.

Our study aims to combine a vertical and horizontal approach in examining potential social inequalities in early onset T2D in the working population. Specifically, we aim to:

1. examine socioeconomic inequalities in early onset T2D in the working population as depicted by educational level and income among nine occupational sectors.
2. define vulnerable subgroups with respect to early onset T2D in the working population while considering both horizontal (occupational sector) and vertical (income and education) dimensions of social stratification.

## Methods

### Data

The study used data from the largest statutory health insurance provider in Lower Saxony, Germany, known as “Allgemeine Ortskrankenkasse Niedersachsen” (AOKN). Health insurance is mandatory for all residents in Germany, and AOKN insures roughly one-third of the population in Lower Saxony, which amounts to approximately 3 million men and women^52^. In the German statutory health insurance system, insurance premiums are based on individual income, while medical services are provided equally to all insured individuals. The dataset includes all inpatient and outpatient diagnoses, classified according to the German-modified tenth version of the International Classification of Diseases (ICD-10-GM), as well as all recorded medical treatments and prescribed medications. Additionally, the dataset includes socioeconomic information such as education, income, and occupation, as reported by employers who are legally required to provide certain information for the social security system. The dataset spans the years 2005 to 2019. The study was done using data for the most recent year available, 2019. The study involved AOKN statutory insured working individuals aged 18 to 40 years.

#### Definition of early onset T2D

The definition of T2D followed an approach similar to that used in previous research with the same dataset^5,14,40,41^. Individuals aged 18–40 years insured for more than one quarter in 2019 were classified as having prevalent T2D if they had valid T2D diagnoses in at least two quarters. However, this condition was not applied to those insured for only one quarter.

The ICD-10-GM codes for Diabetes mellitus fall within the range of “E10” to “E14,” with codes between “10” and “14” indicating different types of diabetes. The code “E11” refers to T2D. However, some inconsistencies were present, as different diabetes codes were occasionally assigned to the same individual. To address this, the study applied specific criteria to validate T2D diagnoses. A T2D diagnosis was considered valid if one of the following conditions was met:

- Among the coded “E” codes, “E11” was the most frequently coded, or
- “E14,” which refers to “unspecified type of diabetes,” was the most frequently coded (as T2D accounts for approximately 90% of diabetes cases), or
- “E10,” which corresponds to T1D, was the most frequently coded, but no insulin prescriptions were coded (since T1D always requires insulin treatment).

#### Occupational sector

Occupations were categorized based on the relevant occupational sectors as defined by the latest classification of occupations (KldB2010), issued by the German Federal Employment Agency in 2010^63^. The KldB2010 classification provides a more detailed structure and better alignment with the international standard classification of occupations (ISCO) than earlier versions. The classification contains 5-digit codes, representing over 1280 occupations divided into 10 major occupational sectors. These sectors are: “Military”, “Agriculture”, “Extraction of raw material, production and manufacturing”, “Construction, architecture, measuring and building technology”, “Natural sciences, geography, information”, “Transport, logistics, protection and security”, “Commercial, trade, distribution and tourism”, “Corporate organization, accounting, law and administration”, “Health sector, social work, teaching & education” and “Humanities, culture and design”. The “Military” sector was excluded from the analyses due to the very small sample size, as healthcare costs for individuals in this sector are usually covered by federal aid (Beihilfe) or private insurance. For individuals working in multiple sectors in 2019, the sector with the longest duration was considered for the analysis.

#### Education and income

Income in the AOKN database is categorized based on its proportion of the average German annual income (AGI), which represents the pre-tax salary of employed individuals as reported annually by the Federal Statistical Office of Germany (Statistisches Bundesamt). Income was classified based on how much it deviated from the AGI. It was categorized in the study into three levels: low (< 60% of the AGI), middle (60–80% of the AGI), and higher (> 80% of the AGI). If individuals had several income levels during the year, the highest income level was considered. In 2019, which is the year of the study, the AGI was 39,301 €^64^. Based on the thresholds set in this study, the corresponding numeric income for the three levels is as follows: Low: <23,581 €; Middle: 23,581 € − 31,441 €; Higher: >31,441 €. There are several reasons for using this approach. By using annual averages, it becomes possible to analyze social gradients across different outcomes. If the income distribution in the study sample does not align with the general population distribution, using unique cut-off values for the sample could hinder comparisons across studies. Since the social structure of the AOKN differs somewhat from that of Germany as a whole^61^, specific income cut-offs may not match national figures. In addition, since in the AOKN population high-income groups are underrepresented^61^, higher threshold for the upper income level could result in a skewed distribution. Therefore, the threshold for the “higher” income category was set at > 80% of the AGI. Nevertheless, the AGI corresponds to the income in terms of the average salary of employed individuals and is thus higher than the average individual annual income in general. This procedure was applied in earlier papers with the present dataset^33^ .

Education was classified based on the highest school-leaving certificate achieved, and represented in terms of years of schooling. The three levels are: low: ≤9 years of schooling (equivalent to the German Hauptschulabschluss or no school diploma), middle: 10 years of schooling (equivalent to the German Realschulabschluss), and high: 12–13 years of schooling (equivalent to the German Abitur, similar to a high school diploma).

#### Statistical analyses

The year prevalence rates of T2D across the various occupational sectors were illustrated using predicted probabilities derived from logistic regression analyses. Predicted probabilities estimate prevalence while accounting for adjusted covariates^65^. First, separate logistic regression models were applied for each of the nine occupational sectors and for the two socioeconomic status variables income and education to display potential income and education differences in T2D prevalence *(vertical inequalities)* among the various occupational sectors *(horizontal inequalities).* In all logistic regression models, the dependent variable was the binary “prevalent T2D” (0 = “no”, 1 = “yes”) with income or education as the main independent variables. Both were added to the models in the form described above with “high” serving as reference category. All models were adjusted for age, gender and number of days insured. The latter was used as a covariate in order to adjust for potential censoring, since not all individuals were insured for the same number of days in 2019. Second, predicted probabilities for T2D were then estimated using the “margins at means” command adjusting for the mean values of all covariates. Therefore, the predicted probabilities in the analyses represent the adjusted year T2D prevalence rates for 2019, taking into account potential confounding effects of age, gender and number of insured days.

In this study, we did not stratify the analyses by gender mainly due to case number limitations since we are dealing with a relatively rare outcome and many subgroups for analyses. The primary focus was not on highlighting gender differences which has been extensively examined in previous studies using the same database^31,43^. Additionally, conducting gender-stratified analyses was beyond the study’s scope in terms of feasibility and clarity of presenting the results. However, to consider potential strong gender influences on the risk of prevalent early onset T2D among sectors and education and income groups while maintaining focus and clarity od results, we adjusted for gender in all models and added double sided interaction terms of gender and SES.

The second phase of the analyses involved using the decision tree method to explore the intersectionality of vertical and horizontal inequalities. This method illustrates how independent variables interact and provides a hierarchical representation of their influence on the outcome variable. Factors that account for the most variance in the outcome are displayed as the first split in the decision tree, followed by additional factors that may significantly impact the outcome through interaction effects with the first split-variable. The analysis employed the “Chi-squared Automatic Interaction Detection” (CHAID) method, which selects the independent variable with the strongest correlation to the dependent variable at each step. If no significant differences exist among certain categories of an independent variable, they are merged. In the regression tree analysis applied, the outcome variable was T2D in its binary form. Independent variables added to the model for which intersectionality was examined were occupational sector (nine categories), income (three categories) and education (three categories). Age in years and gender were adjusted for in the model. The significance threshold for splitting nodes and merging categories was set at *p* < 0.05. For decision tree construction, a minimum of 100 cases was required for parent nodes and 50 for child nodes, with a maximum of three levels for child nodes.

Statistical analyses were conducted using STATA version 16.0 and SPSS version 25.

## Results

The study population ranged between 7855 and 88,667 individuals among the different occupational sectors (*N* = 365,059). The mean age among the sectors was quite similar, ranging between 29 and 31 years. A few occupational sectors were women-dominated such as the sector of “Health, social work, teaching & education”, while men represented the majority of other occupational sectors such as “Construction, architecture, measuring and building technology” and “Transport, logistics, protection and security”. Further detailed sociodemographic characteristics are presented in Table 1.

**Fig. 1:**
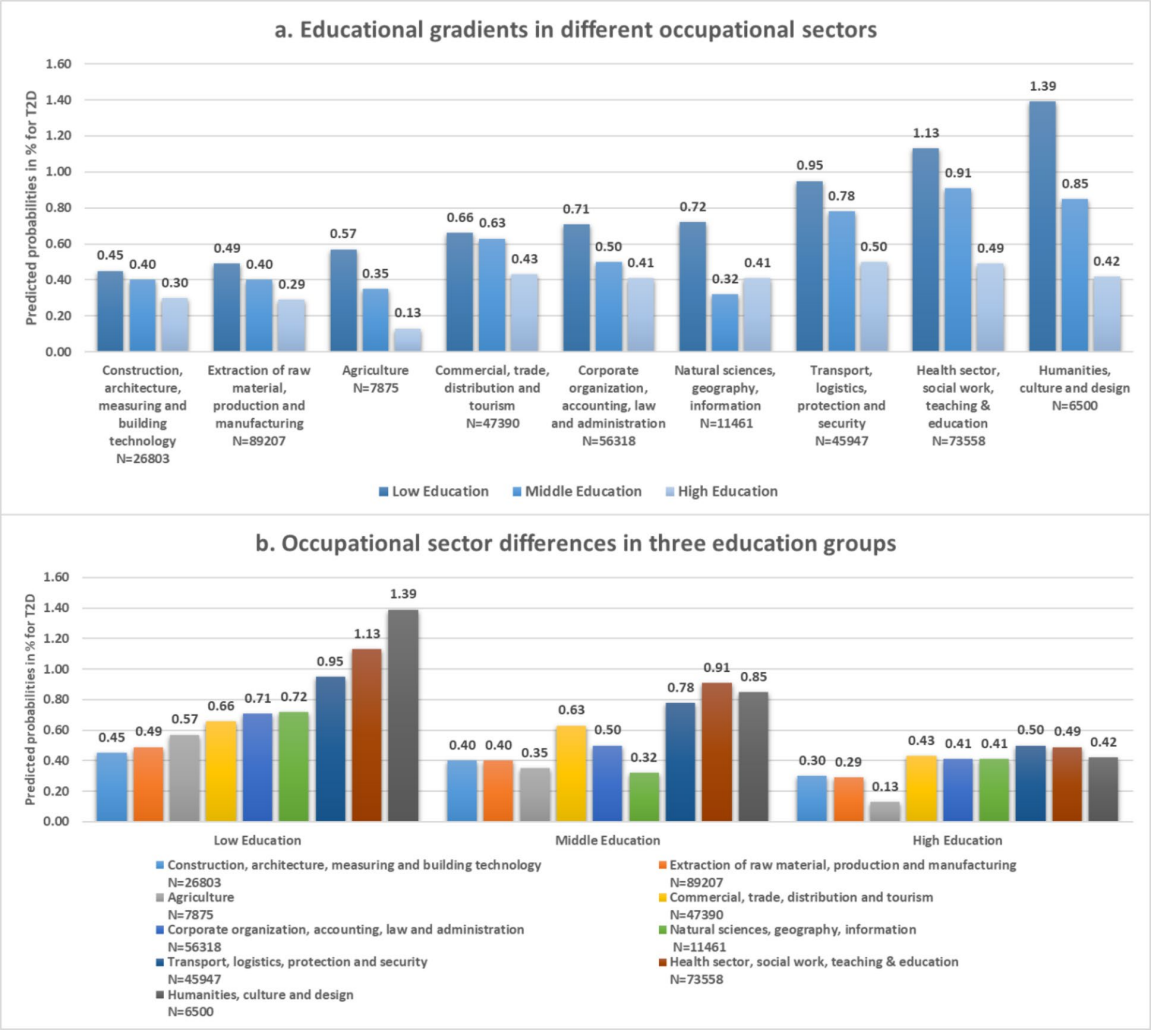
Predicted probabilities for early onset T2D in nine occupational sectors stratified by school educational level, displayed as (**a**) educational gradients in different occupational sectors, and (**b**) occupational sector differences in the three education groups. All models adjusted for age, gender, insurance duration and the interaction effect of gender and education. Source of data: AOKN.

Figure 1a displays educational gradients in early onset T2D among occupational sectors. Gradients exist in favor of higher educational levels among several of the occupational sectors. However, the extent of these gradients vary among different occupational sectors, representing varying levels of the significance of the level of school education in determining the risk of prevalent T2D in different occupational contexts. For example, education did not show a significant effect on determining the odds of prevalent T2D in the sectors like “Construction, architecture, measuring and building technology” and “Natural sciences, geography, information”. In other sectors, education showed a strongly significant effect, which was the highest in the sectors “Corporate organization, accounting, law and administration” OR = 1.28 (CI:0.59 ; 1.97) and “Health sector, social work, teaching & education” OR = 1.17 (CI:0.57 ; 1.77) (Table 2). Moreover, predicted probabilities displayed in Fig. 1b illustrate that within the same level of education, be it low, middle or high, pronounced differences in predicted probabilities among the different occupational sectors exist, illustrating the simultaneous existence of vertical and horizontal inequalities in T2D in the employed population. For example, in the low education group, the predicted probabilities among the different sectors ranged between 0.45% (Construction, architecture, measuring and building technology) and 1.39% (Humanities, culture and design), while they ranged between 0.13% (Agriculture) and 0.50% (Transport, logistics, protection and security) in the high education group.

**Table 1:** Population demographic characteristics by occupational sector. Source of data: AOKN.

	*N*	Age M(Sd)	% Female	T2D (%)	Education (%)			Income (%)		
					Low	Middle	High	Low	Middle	High
Occupational sector										
Agriculture	7855	29 (6)	25.51	0.51	36.85	46.84	16.31	43.33	28.45	28.22
Extraction of raw material, production and manufacturing	88,667	30 (6)	11.89	0.61	28.56	55.12	16.31	24.26	20.14	55.60
Construction, architecture, measuring and building technology	26,667	30 (6)	5.61	0.51	41.06	46.94	12.00	20.74	18.43	60.83
Natural sciences, geography, information	11,412	30 (6)	22.04	0.57	7.12	38.11	54.77	19.30	11.00	69.69
Transport, logistics, protection and security	45,441	31 (6)	22.39	1.10	41.55	44.54	13.91	35.34	31.33	33.33
Commercial, trade, distribution and tourism	47,172	29 (6)	62.68	0.71	21.69	54.43	23.87	57.11	20.22	22.67
Corporate organization, accounting, law and administration	56,057	30 (6)	70.22	0.59	3.07	42.46	54.47	33.88	17.94	48.18
Health sector, social work, teaching & education	72,890	30 (6)	83.37	0.91	9.50	54.76	35.73	43.69	20.26	36.05
Humanities, culture and design	6469	30 (5)	59.86	0.75	5.52	31.36	63.12	31.64	20.01	48.36

**Table 2:** Education and income inequalities in different occupational sectors analyzed by means of logistic regression analyses, adjusting for gender, age and number of insured days. Source of data: AOKN.

	*N*	Education; Reference: High					Income; Reference: High				
		Middle		Low			Middle		Low		
		OR	95% CI	OR	95% CI	Pseudo R2	OR	95% CI	OR	95% CI	Pseudo R2
***Occupational sector***											
Agriculture	7875	0.78	−1.31 ; 2.88	1.59	−0.43 ; 3.61	0.05	−0.22	−1.19 ; 0.75	0.08	−0.87 ; 1.02	0.03
Extraction of raw material, production and manufacturing	89,207	0.31	−0.01 ; 0.63	0.49*	0.16 ; 0.82	0.06	0.13	−0.11 ; 0.36	0.29	−0.00 ; 0.59	0.06
Construction, architecture, measuring and building technology	26,803	0.28	−0.39 ; 0.95	0.32	−0.35 ; 0.99	0.04	0.23	−0.19 ; 0.65	−0.19	−0.81 ; 0.44	0.04
Natural sciences, geography, information	11,461	−0.08	−0.69 ; 0.53	0.63	−0.11 ; 1.36	0.06	0.75*	0.02 ; 1.47	−0.56	−2.02 ; 0.89	0.06
Transport, logistics, protection and security	45,947	0.54*	0.11 ; 0.97	0.78**	0.35 ; 1.20	0.05	0.36**	0.14 ; 0.60	0.17	−0.11 ; 0.45	0.05
Commercial, trade, distribution and tourism	47,390	0.10	−0.37 ; 0.57	0.22	−0.34 ; 0.79	0.03	0.27	−0.22 ; 0.76	0.17	−0.29 ; 0.63	0.03
Corporate organization, accounting, law and administration	56,318	0.47	−0.01 ; 0.94	1.28**	0.59 ; 1.97	0.04	0.55	−0.04 ; 1.14	0.05	−0.20 ; 1.12	0.04
Health sector, social work, teaching & education	73,558	0.69*	0.21 ; 1.17	1.17**	0.57 ; 1.77	0.04	0.27	−0.30 ; 0.83	0.63*	0.15 ; 1.10	0.03
Humanities, culture and design	6500	0.88	−0.18 ; 1.95	1.30	−0.09 ; 2.70	0.05	0.58	−0.52 ; 1.68	0.14	−1.18 ; 1.45	0.04

*p < 0.05; **p < 0.001

**Fig. 2:**
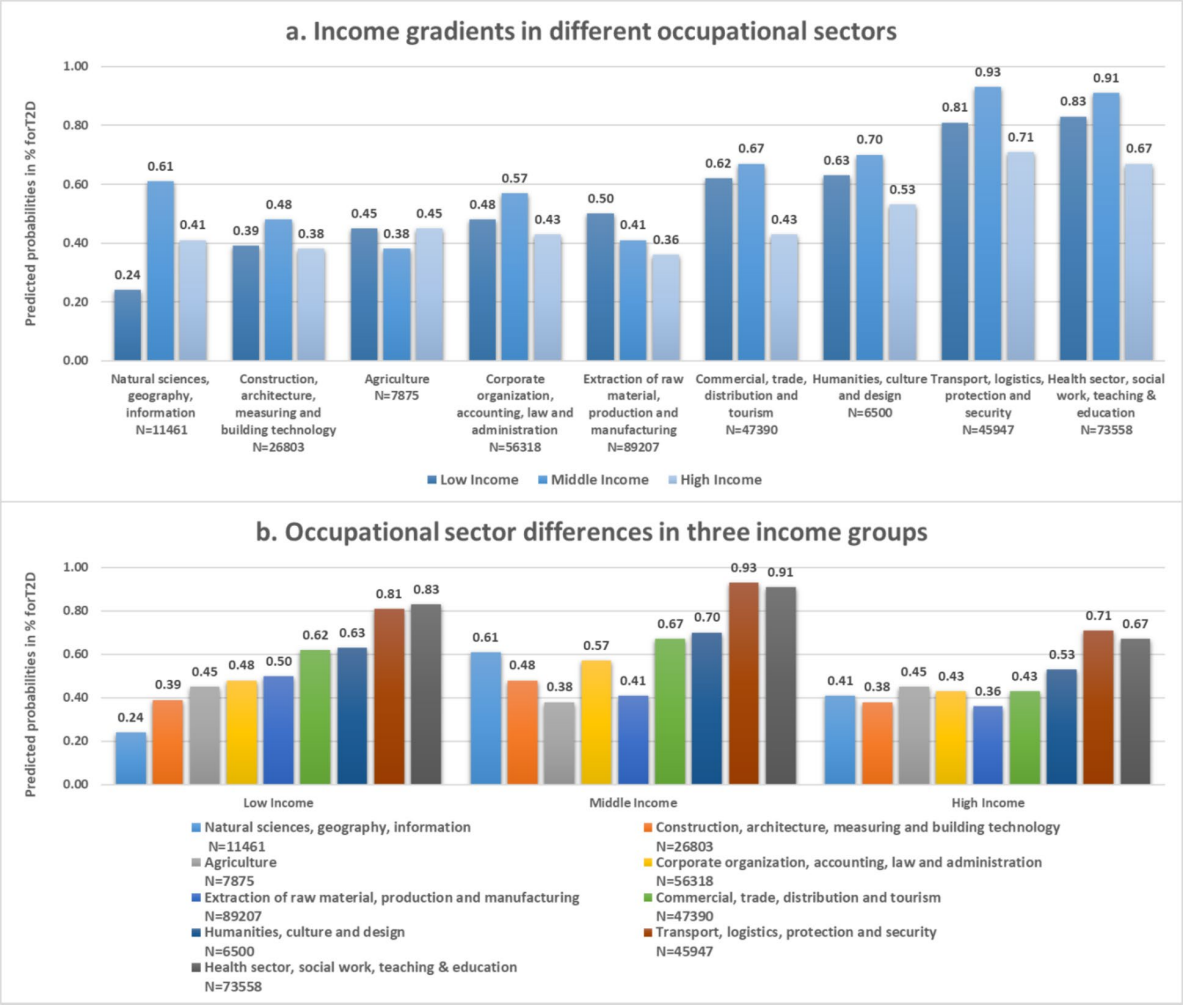
Predicted probabilities for early onset T2D in nine occupational sectors stratified income level, displayed as (**a**) income gradients in different occupational sectors, and (**b**) occupational sector differences in the three income groups. All models adjusted for age, gender, insurance duration and the interaction effect of gender and income. Source of data: AOKN.

With respect to income level, vertical differences have been also observed horizontally among the different occupational sectors (Fig. 2), but to a lower extent compared to education. In three of the nine occupational sectors, there were significant income inequalities on T2D prevalence (Fig. 2a; Table 2). For example, significant inequalities were observed for the sectors “Health, social work, teaching and education” OR = 0.63 (CI:0.15–1.10) for the low versus the high income group, and “Natural sciences, geography, information” OR = 0.75 (CI:0.02–1.47) for the middle versus the high income group (Table 2).

**Fig. 3:**
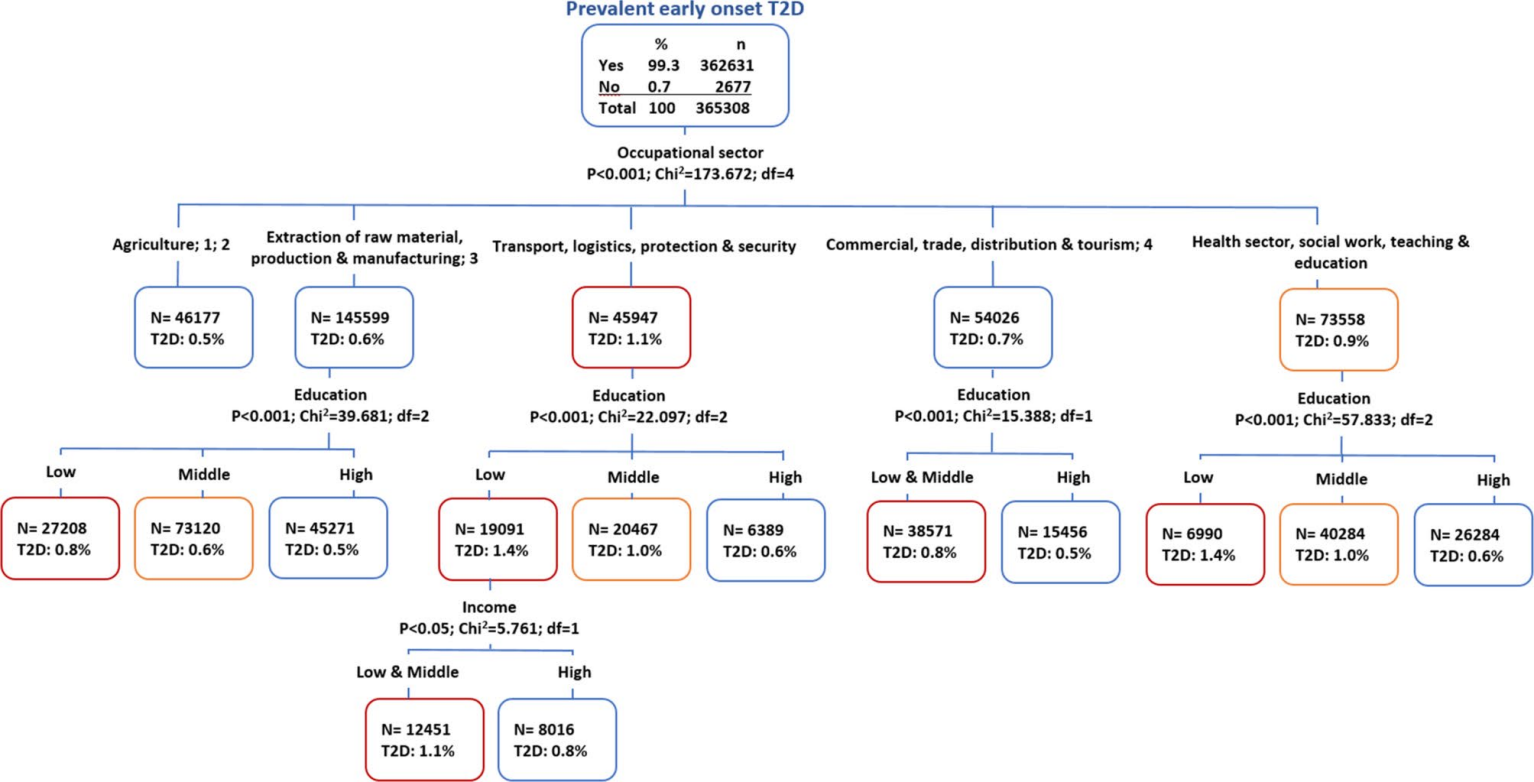
Regression tree illustrating intersectionality between vertical (education and income) and horizontal (occupational sector) inequalities in early onset T2D in the workforce. *1: Construction*,* architecture*,* measuring & building technology; 2: Natural sciences*,* geography*,* information; 3: Corporate organization*,* accounting*,* law & administration; 4: Humanities*,* culture & design.* Orange frame: *p* < 0.05; Red frame: *p* < 0.001. Source of data: AOKN.

The regression tree analyses displayed different vulnerability levels for the different occupational sectors with respect to T2D. At the first level, the regression tree analyses displayed that the occupational sector was the variable that mostly defined vulnerable groups (*p* < 0.001). The regression tree displayed five splits of occupational sectors, with the sectors “Transport, logistics, protection and security” (1.1%) and “Health, social work, teaching and education” (0.9%) as the most vulnerable. At the second level, education was the factor defining vulnerability levels in four of the five occupational group child nodes displayed, with clear educational level gradients in favor of the higher education groups in all of them. The highest vulnerabilities were observed for the low income groups of the sectors “Transport, logistics, protection and security” and “Health, social work, teaching and education”, with 1.4% of individuals in these groups having prevalent T2D in 2019 (*p* < 0.001). At the third level, income appeared to define vulnerability only in the middle education group of the sector “Transport, logistics, protection and security”, with low and middle income being significantly more vulnerable (rate = 1.1%, *p* < 0.05) compared to the high income group. While the model also included gender and age, no splits with respect to these two variables were observed among the three displayed levels of vulnerability (Fig. 3).

## Discussion

The study investigated social inequalities in early onset T2D in working individuals by combining vertical and horizontal stratification of social groups of the young working population. The results indicated that vertical and horizontal inequalities coexist in defining vulnerable groups. Socioeconomic gradients were observed in favor of higher income and education groups. However, income appeared to be less powerful in explaining the risk of T2D prevalence compared to education. The results indicated that within the same level of vertical stratification, i.e. the same level of education or income, pronounced occupational sector differences existed with respect to early onset T2D. The relative vulnerability level of the occupational sectors however partly differed depending on the socioeconomic indicator considered, indicating the importance of simultaneously considering multiple dimensions and measures of socioeconomic status when aiming to examine inequalities. Moreover, the regression tree analysis showed that occupational sector, illustrating the horizontal dimension of inequalities, defined vulnerability levels in early onset T2D better than the vertical dimension, illustrated by education and income. At the second level, education was a highly significant indicator for prevalent early onset T2D among the different occupational sectors. Particularly, individuals with lower levels of education working in the sectors “Transport, logistics, protection and security” and “Health sector, social work, teaching and education” were shown to be the most vulnerable subgroups for prevalent early onset T2D. Our study designates the importance of following the approach of combining the *vertical* and the *horizontal* dimensions of social stratification when aiming to examine social inequalities in health in the working population, as both dimensions appeared to determine vulnerability levels independently. As to our knowledge, no studies exist that have pursued this approach, the results of this study highlight the importance of following this approach in future studies aiming to examine vulnerable social groups for other outcomes and populations.

Socioeconomic inequalities in T2D have been well established in the literature^53,54 ^even among the working population^5^. Nevertheless, evidence on occupational inequalities regardless of the underlying effect of the socioeconomic status hierarchy in different occupational contexts was limiting. In this study, the different vulnerability levels of early onset T2D among the different occupational sectors which were observed within all vertical hierarchies of the socioeconomic status, as depicted by the education and income levels, reflect that the work context is an important one in determining the risk profiles of working individuals. Similar to a previous analysis^43^, the occupational sectors “Transport, logistics, protection and security” and “Health sector, social work, teaching and education” were shown to be among the most vulnerable. This study however added that this applies also when stratifying the population by income and education, ruling out an explicit effect of the vertical hierarchy of the socioeconomic status on defining risk profiles of individuals working in specific occupations. For example, it can be argued that the occupational sector of “Transport, logistics, protection and security” is a vulnerable one due to the high proportion of individuals with a lower socioeconomic status^55^. However, the study showed that among individuals in the high education and income groups, predicted probabilities of T2D in individuals working in this sector were the highest compared to the other sectors. Similarly, the occupational sector “Health, social work, teaching and education” remained to be among the most vulnerable in all vertical socioeconomic strata. It can thus be argued that occupational contexts are highly associated with different occupational exposures^56,57^which shape the metabolic risk profiles of individuals. For example, individuals working in the transport sector have prolonged sitting times and less possibility for being active during work, posing them to higher risk levels for T2D. On the other hand, while individuals working in the health sector such as hospital health care providers can be physically active during work, stress, sleep deprivation and shift work which can be coupled with inadequate nutritional behavior can be associated with higher risks for T2D in this sector. In fact, there is sufficient evidence on the existence of work environment-specific exposures such as inadequate physical activity^37^, limited resources needed to enhance health promoting behavior^27^, stress^29,38^, shift work^28,39 ^and exposure to chemicals^40^ that can be associated with a high risk of T2D regardless of the socioeconomic resources individuals possess. Our study showed that intersectionality between socioeconomic status, especially educational level, and occupational sector exist in defining vulnerable groups with respect to early onset T2D prevalence, but occupational sector outweighs the income and education level in defining vulnerability levels. Thus, working individuals would for sure benefit from occupational-context tailored prevention strategies rather than a “one size fits all” approach.

The study also displayed that while the two examined vertical socioeconomic status measures had significant effects in determining inequalities in early onset T2D prevalence, education appeared to be a more pronounced indicator than income. In addition, education was strongly associated with defining vulnerability levels within the occupational sectors. Previous studies have reported similar results on the relatively high explanatory power of education compared to other socioeconomic indicators in the context of T2D. Proposed mediating factors for the association between education and T2D is health literacy and risk factors^5,58^, which are also more common in individuals with lower education. In this study, the relatively high explanatory power of education on the risk of prevalent T2D has been confirmed also for the young working population and among the different occupational sectors, further highlighting the importance of educational level in determining and addressing inequalities in T2D.

The results of the study also indicate that the intersection of vertical and horizontal inequalities might create differential exposures to risk factors that may accelerate adverse health effects and onset of disease^59^. The relationship between these factors is not necessarily merely additive but could rather be synergistic, with, for example, certain occupational sectors exacerbating the effects of low education or income on early onset T2D. For example, as was shown in the regression tree analyses, individuals with low education levels working in sectors like “Transport, logistics, protection, and security” or “Health, social work, teaching, and education” are particularly vulnerable. These sectors often involve high levels of physical stress, irregular working hours, and lower job control which are associated with higher levels of cortisol, irregular eating patterns, and poor metabolic regulation—all potential risk factors for early T2D onset. Individuals with high levels of education working in these sectors might be affected by these stressors to a lower extent compared to their low education counterparts due to better health literacy and health management skills that also shape lifestyle and health behavior. Thus, a unique set of risk exposures might be created among vulnerable groups that shape the timing and severity of disease.

Individuals with early onset T2D are a particularly high-risk group, making vertical and horizontal inequalities even more relevant. In contrast to the general onset of T2D, which is often studied in relation to long-term health outcomes; early onset is marked by accelerated biological changes and potentially irreversible damage to organ systems. The intertwined mechanisms of factors associated with vertical and horizontal inequalities described above might drive the *early* onset of T2D in working individuals. The combined effect of early exposure to unhealthy working conditions and socioeconomic stress may significantly shorten the window for preventive interventions and accelerate the onset of disease. Hence, working individuals could benefit from policies that include better healthcare access, tailored workplace wellness programs, and health education programs which may alleviate the cumulative burden of risk factors in the most vulnerable sectors.

### Strengths and limitations

This is the first study to consider multidimensional inequalities in early onset T2D in the working population using the vertical-horizontal approach. Early onset T2D is an alarmingly growing outcome with an increasing public health concern and economic relevance. Yet, it still concerns a relatively low case number since the average age at onset for T2D is around 45 years^60^. The large dataset of AOKN made it possible to apply a multidimensional stratification while examining early onset T2D in the employed population despite relatively low case numbers. Still, the study has some limitations. First, since our aim was to examine whether vertical and horizontal inequalities simultaneously exist, we considered broad occupational sectors rather than specific occupations in order to provide an overview on multidimensional T2D inequalities in the occupational context. Future studies should consider a more detailed stratification of occupational groups in order to tackle specific occupational vulnerabilities in early onset T2D. Second, the AOKN is a statutory health insurance provider in Lower Saxony. Some specific occupational groups might be under-represented in the data due to having other convenient insurance options, and one occupational sector, mainly “Military” was not represented in the data. Moreover, the socioeconomic structure of the AOKN differs somewhat from that of the general population in Germany^61^, which might affect the generalizability of the results. The socioeconomic stratification applied in the analyses, however, weakens potential selection effects associated with specific socioeconomic groups being over or under represented. Third, we did not stratify the analyses by gender mainly due to case number limitations. Nevertheless, examining gender differences in early onset T2D was not the focus of the study. However, since other studies displayed gender occupational differences in health outcomes^31,43,62^, future more focused studies should consider a gender stratified analysis. In addition, While the use of secondary data is a significant strength of the study, minimizing sources of selection bias commonly seen in surveys (such as recall, morbidity, and reporting bias), a limitation is the potential for coding errors, including double or incorrect coding. We took steps to apply inclusion criteria that would capture valid diagnoses, such as considering insulin prescriptions to reduce the risk of erroneously including type 1 diabetes cases. However, it is important to acknowledge that diagnostic errors cannot be entirely ruled out.

## Conclusion

Vertical and horizontal socioeconomic inequalities exist in early onset T2D in the employed population, and both intersect in determining morbidity. With our study, we aimed to introduce an important and often neglected dimension of inequality into social epidemiology. Viewed from the one side, it was shown that education and income-related inequalities exist within different occupational sectors. Viewed from the other side, it was shown that pronounced occupational sector differences exist within the same levels of education and income. The results depict occupation-specific working conditions and specific expositions associated with disease risks. In the first place, occupational sector mostly determined vulnerability for prevalent early onset T2D, followed by level of education. The simultaneous consideration of vertical and horizontal dimensions of inequalities is therefore essential to determine vulnerable groups in the working population. Future studies should follow this approach while considering other populations and health outcomes that are known to be dependent on behavior or on working conditions. Cardiovascular diseases and musculoskeletal disorders are potential candidates. We defined vulnerable groups with respect to early onset T2D in the working population of which individuals with low education working in the sectors “Transport, logistics, protection and security” and “Health, social work, teaching and education” are among the most affected. A more detailed examination of the occupational groups underlying these sectors is needed in order to define more specific vulnerable groups for which T2D prevention interventions should be tailored.

### Ethical approval

This study did not require ethical approval since it utilized a pre-existing claims dataset that was created as part of AOKN’s routine administrative activities. The scientific use of this data is regulated by German law within the German Social Code, “Sozialgesetzbuch.” Permission to use the data for research purposes was granted by the data protection officer of the Local Statutory Health Insurance of AOKN.

## Data Availability

The data underlying this study belong to the Allgemeine Ortskrankenkasse Niedersachsen (AOKN-General Local Health Insurance of Lower Saxony). The data are not publically available due to protection of data privacy of the insured individuals by the AOKN. Interested researchers can send data access requests to Dr. Jona Stahmeyer at the AOKN using the following e-mail address: Jona.Stahmeyer@aok.nds.de. The authors did not have any special access privilege.
